# Comparison of bracket bond strength to etched and unetched enamel under dry and wet conditions using Fuji Ortho LC glass-ionomer

**DOI:** 10.15171/joddd.2017.006

**Published:** 2017-03-15

**Authors:** Masoud Feizbakhsh, Farzin Aslani, Naghme Gharizadeh, Mojtaba Heidarizadeh

**Affiliations:** ^1^Assistant Professor, Department of Orthodontics, School of Dentistry, Isfahan Branch, Islamic Azad University, Khorasgan, Iran; ^2^Orthodontics Surgery Fellowship, Department of Orthodontics, School of Dentistry, Shahid Beheshti University of Medical Sciences, Tehran, Iran; ^3^Assistant Professor, Department of Orthodontics, School of Dentistry, Ahvaz University of Medical Science, Ahvaz, Iran; ^4^DDS, Dental School, Ahvaz University of Medical Sciences, Ahvaz, Iran

**Keywords:** Acid etching, bond strength, Fuji Ortho LC glass-ionomer, moisture condition

## Abstract

***Background.*** Acid etching prior to orthodontic bracket bonding may result in enamel wear or cracks following bracket removal. The manufacturer of Fuji Ortho LC glass-ionomer (GI) claims that it can bond brackets to wet unetched enamel. This study aimed to compare the bracket bond strength to etched and unetched enamel under dry and wet conditions.

***Methods.*** In this in vitro study, 60 intact premolar teeth were randomly assigned to 6 groups (etched and dried, etched and moistened with distilled water, etched and moistened with saliva, unetched and dried, unetched and moistened with water, unetched and moistened with saliva). In all the groups, Leon 4 brackets were bonded to the enamel using Fuji Ortho LC GI. The teeth were immersed in distilled water at 37°C for 24 hours and subjected to shear loads at a crosshead speed of 5 mm/min in a Zwick machine for bond strength testing. Data were analyzed with ANOVA, Tukey test and independent t-test.

***Results***. The mean bond strength values in groups 1 (etched, dry), 2 (etched, moistened with water), 3 (etched, moistened with saliva), 4 (unetched, dry), 5 (unetched, moistened with water) and 6 (unetched, moistened with saliva) were 21.86, 16.46, 10.49, 8.12, 9.15 and 9.52 MPa, respectively. Significant differences in bond strength were detected between groups 1 and 2 and all the other groups (P < 0.05), with no significant difference between groups 1 and 2 (P > 0.05).

***Conclusion.*** Fuji Ortho LC GI provided adequate bond strength between brackets and enamel. To acquire higher bond strength, brackets must be bonded to etched and dried enamel.

## Introduction

Bracket bonding to tooth structure replaced banding and revolutionized the contemporary orthodontics. Composite resins are commonly used for bracket bonding to enamel. However, they have high technical sensitivity. A good composite resin bond requires a completely dry surface throughout the process of bonding; in addition, the enamel surface must be etched for 15‒60 seconds. In this regard, patient cooperation and the time required are important issues that need to be taken into account.^1^

Due to the fluoride release potential and high compatibility with enamel and dentin, GI cement is an important dental material in restorative dentistry. The newly introduced GI cements provide good color match with teeth due to their different shades. Furthermore, the new GI cements have higher translucency and a suitable finished surface due to their high content of modified resin. However, they do not have adequate opacity, as do composite resins. In the process of enamel etching, more than 10% of the enamel surface is eliminated^2^and crack and wear also occur after bracket removal.^3^Moreover, the process of resin removal is time-consuming and costly and accumulation of plaque around the bracket leads to demineralization of enamel, and white spot lesions may consequently develop.^4^Acid etching creates a rough and highly irregular enamel surface with increased surface free energy. By application of a resin-based flowable material on an irregular, etched surface, resin penetrates into the surface and infiltration is intensified by the capillary action.^2^

The above-mentioned problems greatly decrease by the application of light-cured reinforced GI cement (Fuji Ortho LC). This adhesive cement releases fluoride and prevents enamel demineralization. It does not require isolation or enamel etching prior to bonding.^5^However, it provides lower bond strength to the enamel compared to composite resins. This study aimed to assess the bond strength of Fuji Ortho LC GI to etched and unetched enamel in dry and wet conditions.

## Methods

In this experimental study, 60 extracted premolar teeth with sound buccal surfaces and no carious lesions, cracks or fractures were selected. The teeth had not received any chemical treatment prior to extraction. The collected teeth were stored in 0.2% thymol solution and retrieved from the solution 24 hours prior to testing. After cleaning, the teeth were immersed in distilled water at room temperature.

A previously designed cylindrical metal mold was used for fabrication of specimens. The internal surface of the mold was coated with a layer of Vaseline and filled with auto-polymerizing acrylic resin (Bayer, Pittsburgh, USA). The specimens were mounted in acrylic resin in such a way that the height of contour of the teeth in the buccal surface was in contact with the surveyor blade. From the mesiodistal dimension, the longitudinal axis of the teeth was perpendicular to the horizontal plane. The heat produced from the polymerization of acrylic resin was controlled by placing the mold in water.

The surface of specimens was cleaned by a low-speed rotary instrument and a prophylactic brush (oil-free) for 10 seconds. The surface of all the specimens was then washed with oil-free water and air spray and dried with oil-free air spray. The teeth were randomly assigned to 6 groups as follows:

Group 1 (application of Fuji Ortho LC GI on etched and dried enamel surfaces before bonding): The specimens were etched with 37% phosphoric acid for 30 seconds, rinsed under running water for 15 seconds, rinsed again with oil-free air and water spray for 15 seconds and completely air-dried with oil-free spray for 20 seconds. Fuji Ortho LC GI (174-8585, GC Corporation, Tokyo) was prepared and applied to the bracket mesh and the brackets were placed on the teeth. The bracket slot was adjusted parallel to the horizontal plane and the surveyor blade was positioned perpendicular to the bracket base. The blade of the bond strength tester was vertical to the bracket base. One scoop of powder and one drop of liquid were placed on a mixing pad. The powder was divided into two parts. The first part was mixed with all the liquid for 10 seconds. The remaining powder was also added and mixed for 10‒15 seconds. The bracket was compressed over the enamel surface by placing a special blade in the bracket slot to achieve minimum adhesive thickness beneath the bracket. Excess glass-ionomer was removed by the sharp tip of an explorer. The specimens were cured from the mesial, distal, occlusal and gingival aspects for 10 seconds each, using a light-curing unit (Coltolux 50, Coltene).

Group 2 (application of Fuji Ortho LC on etched enamel surfaces moistened with distilled water before bonding): All the steps were performed similar to those in group 1. The only difference was that the buccal surfaces of the teeth were moistened with distilled water using a microbrush.

Group 3 (application of Fuji Ortho LC on etched enamel surfaces moistened with saliva before bonding): The specimens were prepared similar to that in group 1 and the buccal surfaces of the teeth were moistened with saliva using a microbrush.

Group 4: Fuji Ortho LC GI was applied on unetchedenamel surfaces. The teeth were dried before bonding.

Group 5: Fuji Ortho LC was applied on unetched enamel surfaces and the teeth were moistened with distilled water before bonding.

Group 6: Fuji Ortho LC was applied on unetched enamel surfaces and the teeth were moistened with saliva prior to bonding.

**Figure 1.**
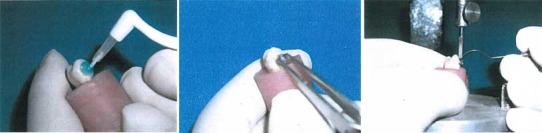
Preparation of samples for loading force.

All the specimens were stored in distilled water in an incubator at 37°C for 24 hours. To calculate the debonding force, a universal testing machine was used (Zwick: Z600EWN: 1484136, Zwick GmbH & Co. KG at Ulm, Germany). This device has two jaws (one fixed and one removable). Metal cylinders were placed in their specific location in the fixed jaw of the device. A special sharp blade was also designed for load application. Each specimen was fixed in its place in the Zwick machine and shear load was applied at a crosshead speed of 5 mm/min (Figure 1). The load was applied to the bracket base at the base‒wing joint. The blade applied load vertically to the bracket base. Zwick machine digitally displayed the load required for bracket debonding. The load required for bracket debonding (in Newtons) was divided by the surface area of the bracket mesh base to calculate the bond strength value in MPa. The bond strength values in different groups were analyzed using ANOVA. Pairwise comparisons were performed with Student’s t-test.

## Results

The debonding loads in etched and unetched specimens were 140.87 ± 64.96 and 77.3±45.06 N, respectively; this value was significantly greater in the etched group (P < 0.001). Moreover, the bond strengths in etched and unetched specimens were 16.27 ± 7.5 MPa and 8.93 ± 5.2 MPa, respectively, with a significant difference between the two groups (P < 0.001).

The highest mean debonding load (189.3 MPa) was recorded in group 1 and the lowest (70.3 MPa) was noted in group 4. Significant differences were noted in terms of debonding loads between groups 1 and 2 and all the other groups (P<0.001); but there was no significant difference between groups 1 and 2 (Table 1).

**Table 1:** The mean and standard deviation of debonding load in study groups (Newton)

**Group**	**The method used**	**Mean**	**Standard deviation**
**1**	Application of Fuji Ortho LC on etched and dried enamel surfaces before bonding	189.3	49.83
**2**	Application of Fuji Ortho LC on etched enamel surfaces moistened with distilled water before bonding	142.5	64.02
**3**	Application of Fuji Ortho LC on etched enamel surfaces moistened with saliva before bonding	90.8	40.27
**4**	Application of Fuji Ortho LC on unetched and dried enamel surfaces before bonding	70.3	53.76
**5**	Application of Fuji Ortho LC on unetched enamel surfaces moistened with distilled water before bonding	79.2	49.73
**6**	Application of Fuji Ortho LC on unetched enamel surfaces moistened with saliva before bonding	82.4	43.03

In addition, the highest bond strength (21.86 N) was recorded in group 1 (application of GI on etched and dried enamel surfaces before bonding) and the lowest (8.12 N) was recorded in group 4 (application of GI on unetched and dried enamel surfaces before bonding). There was no significant difference between groups 1 and 2 (P > 0.05). Group 1 exhibited significant differences from group 3 (P = 0.003), group 4 (P < 0.001), group 5 (P < 0.001) and group 6 (P < 0.001). Group 2 also exhibited significant differences from group 3 (P = 0.032), group 4 (P = 0.005), group 5 (P = 0.015) and group 6 (P = 0.014) (Table 2). Other groups did not exhibit significant differences from each other.

**Table 2:** The means and standard deviations of bond strengths in the study groups (MPa)

**Group**	**The method used**	**Mean**	**Standard deviation**
**1**	Application of Fuji Ortho LC on etched and dried enamel surfaces before bonding	21.86	5.75
**2**	Application of Fuji Ortho LC on etched enamel surfaces moistened with distilled water before bonding	16.46	7.39
**3**	Application of Fuji Ortho LC on etched enamel surfaces moistened with saliva before bonding	10.49	4.65
**4**	before bonding	8.12	6.21
**5**	Application of Fuji Ortho LC on unetched enamel surfaces moistened with distilled water before bonding	9.15	5.74
**6**	Application of Fuji Ortho LC on unetched enamel surfaces moistened with saliva before bonding	9.52	3.82

## Discussion

The results of the present study showed that the mean debonding load and bond strength values on etched enamel surfaces were significantly higher than those on unetched enamel surfaces. Thus, for bonding Leon brackets with Fuji Ortho LC GI to the tooth surface, etching the enamel surface significantly increases the bond strength. In a study by Bishara et al,^6^ the two groups of etched and unetched teeth exhibited significant differences in bond strength, consistent with our results. Chung et al^7^ also reported similar findings. However, in a study by Kirovski and Madzarova,^8^ the two groups of etched and unetched specimens exhibited no significant difference. The difference between the results of Kirovski and Madzarova^8^and the current study might be attributed to the fact that we tested the shear bond strength in our study while they used the tensile bond strength.

On the other hand, in our study the debonding load in etched and dried specimens was higher than that in etched and moist groups and the mean debonding load in unetched, dried group was lower than that in unetched moist group. This finding is also true for the bond strength values in these groups. It seems that in the etched groups, presence of saliva prevents micromechanical bonding between glass-ionomer and enamel to a great extent due to the deposition of its constituents. However, in the unetched group (with no micron-scale porosities caused by etching), the retention is based on chemical bonding between glass-ionomer and enamel, and presence of moisture, especially saliva, slightly increases the bond strength. Bond strength in this group did not even reach the bond strength value in group 3 (the lowest bond strength among the etched groups). Increased bond strength in group 6 might be due to an increase in calcium ions on the enamel surface adjacent to the saliva in the oral cavity.

On the other hand, since GI bonds to tooth surfaces via chemical mechanisms,^9^ enamel etching might not be necessary for achieving a micromechanical bond.^10^ Another study evaluated and compared the bond strength of enamel prepared with 10% polyacrylic acid for 10 seconds to ceramic and stainless steel brackets using Fuji Ortho LC GI. The results showed that Fuji Ortho LC GI applied to dry enamel yielded similar bond strength values in the two groups of brackets. However, in ceramic brackets with a chemical retention mechanism, composite resin yielded higher bond strength values.^11^

Composite resins are commonly used for direct attachment of orthodontic brackets to enamel.^12^However, acid etching has drawbacks such as calcification of enamel surface and an increase in the risk of enamel fracture during composite debonding. Considering the advantages of GI such as enamel protection by releasing fluoride and less susceptibility to moisture,^13^ GI is an alternative to adhesives for direct bonding of orthodontic brackets.^12^Many studies have compared the bond strength of composite resins and GIs.^14-16^ Reddy et al^14^ showed that the bond strength of glass-ionomer was less than that of composite resin and post-contamination with blood decreased the bond strength of both materials, similar to the findings reported by Yassaei et al.^13^ Summer^15^ showed that conventional adhesive resins conditioned with 37% phosphoric acid had the highest mean shear bond strength and Fuji Ortho LC conditioned with 10% polyacrylic acid had the lowest mean shear bond strength. Rix^16^ showed that all the adhesives (Transbond XT, Fuji Ortho LC, Assure) had acceptable bond strength and the mean bond strength of Transbond XT was greater than that of other groups. Fuji Ortho LC showed greater bond strength than Assure.

It seems that if enamel surfaces are not completely dry, Fuji Ortho LC cement yields higher bond strength. In a study by Cacciafestaet al,^4^ after preparation of enamel surfaces, metal brackets yielded higher bond strength values in the presence of saliva, and both ceramic brackets yielded higher bond strength in the presence of water. In bonding to enamel, enamel surfaces are moistened by the application of the bonding agent and thus higher bond strength values in wet conditions might be related to the presence of 2-hydroxy ethyl methacrylate (HEMA) as the main constituent of Fuji Ortho LC. In other words, a hydrophilic water-soluble monomer is the main component responsible for hydration and infiltration.^17^

Improved bond strength values after applying an etchant on the enamel surfaces is probably due to the fact that the etching process results in significant enamel surface roughness; thus the resin can easily flow over these surfaces. On the other hand, it has been shown that acid etching creates honeycomb structures on enamel surfaces, which increases the mechanical retention.^18^

Etchants remove the organic biofilm with low energy bond to tooth surfaces and selectively etch the enamel surface and cause porosities. On the other hand, it has been shown that 15‒20-μ resin tags are formed at the interface of resin‒etched enamel.^19,20^Moreover, improved bonding following acid etching might be related to the low surface tension of the liquid. As a result, the liquid quickly flows and penetrates into the irregular surface porosities.

Retief^21^ reported that the process of etching increases the wettability of enamel surfaces because of the increased contact between resin and tooth surfaces. Application of acid on enamel surfaces also eliminates the low-energy organic biofilm and selectively etches the mineral phase of the enamel, which significantly increases the accessible areas for the bond to adhesives. Enamel etching significantly improves the contact area between adhesive resins and enamel surfaces. Moreover, insignificant microleakage at the enamel‒resin interface indicates the presence of extra chemical bonds at the interface. If bonding occurs via these forces, the reactive phases significantly increase.^22^At the same time, the Van der Waals and polar forces and the hydrogen bonds all play a role in the bond to enamel and dentin.^23^

Presence of a chemical bond between the GI cement and enamel was confirmed in the current study because all the brackets bonded to enamel surfaces with different surface treatments showed bond strength values within 6‒8 MPa, which is sufficient for clinical applications.^24^ Minimum bond strength values have been shown to be in the range of 6‒8 MPa in the literature.^24^ In addition, maximum bond strength must be lower than the enamel fracture threshold, which has been reported to be 14 MPa.^25^

Thus, when using Fuji Ortho LC GI, the surface should not be necessarily dry and even the enamel preparation step might be skipped.^1^ Thus, use of Fuji Ortho LC GI for bracket bonding saves time, releases fluoride and exerts cariostatic effects. It also reduces the occurrence of white spot lesions next to bracket margins.^26,27^

## Conclusion

Since a 6-MPa bond strength value is clinically acceptable, all the study groups yielded acceptable bond strengths for clinical applications. If higher bond strength is required for bonding of Leon brackets with Fuji Ortho LC GI, the specimens should be etched and dried. If the clinicians prefer not to etch the enamel, complete drying should be prevented and the tooth surface must remain slightly moist.

## Authors’ contributions

The study was planned by MF and NGH. MH carried out the experiments. Manuscript preparation was carried out by FA. All the authors approved the final manuscript.

## Acknowledgments

The authors would like to thank Ahvaz University of Medical Sciences for their financial support of this study.

## Funding

The Dental School of Ahvaz University of Medical Sciences financially supported this study.

## Competing interests

The authors have no competing interests to declare with regards to authorship and/or publication of this article.

## Ethics approval

Not applicable.
