# Erratum to: Comparison of bracket bond strength to etched and unetched enamel under dry and wet conditions using Fuji Ortho LC glass-ionomer

**DOI:** 10.15171/joddd.2017.047

**Published:** 2017-12-13

**Authors:** Masoud Feizbakhsh, Farzin Aslani, Naghme Gharizadeh, Mojtaba Heidarizadeh

**Affiliations:** ^1^Assistant Professor, Department of Orthodontics, School of Dentistry, Isfahan Branch, Islamic Azad University, Khorasgan, Iran; ^2^Orthodontics Surgery Fellowship, Department of Orthodontics, School of Dentistry, Shahid Beheshti University of Medical Sciences, Tehran, Iran; ^3^Assistant Professor, Department of Orthodontics, School of Dentistry, Ahvaz University of Medical Science, Ahvaz, Iran; ^4^DDS, Dental School, Ahvaz University of Medical Sciences, Ahvaz, Iran

In the article entitled “Comparison of bracket bond strength to etched and unetched enamel under dry and wet conditions using Fuji Ortho LC glass-ionomer” which appeared in *J Dent Res Dent Clin Dent Prospect* 2017; 11(1):30-35, the corresponding author’s designation was incorrectly placed. The corresponding author of the article was Dr. Aslani. The journal regrets this error.

